# Mutations in *Drosophila* tRNA processing factors cause phenotypes similar to Pontocerebellar Hypoplasia

**DOI:** 10.1242/bio.058928

**Published:** 2022-03-18

**Authors:** Casey A. Schmidt, Lucy Y. Min, Michelle H. McVay, Joseph D. Giusto, John C. Brown, Harmony R. Salzler, A. Gregory Matera

**Affiliations:** 1 Curriculum in Genetics and Molecular Biology, University of North Carolina, Chapel Hill, NC 27599, USA; 2 Department of Biology, University of North Carolina, Chapel Hill, NC 27599, USA; 3 Integrative Program for Biological and Genome Sciences 27599, University of North Carolina, Chapel Hill, NC 27599, USA; 4 Department of Genetics, University of North Carolina, Chapel Hill, NC 27599, USA; 5 Lineberger Comprehensive Cancer Center, University of North Carolina, Chapel Hill, NC 27599, USA

**Keywords:** RNA processing, Animal models of human disease, Neurodegeneration, tRNA splicing

## Abstract

Mature transfer (t)RNAs are generated by multiple RNA processing events, which can include the excision of intervening sequences. The tRNA splicing endonuclease (TSEN) complex is responsible for cleaving these intron-containing pre-tRNA transcripts. In humans, TSEN copurifies with CLP1, an RNA kinase. Despite extensive work on CLP1, its *in vivo* connection to tRNA splicing remains unclear. Interestingly, mutations in *CLP1* or *TSEN* genes cause neurological diseases in humans that are collectively termed Pontocerebellar Hypoplasia (PCH). In mice, loss of Clp1 kinase activity results in premature death, microcephaly and progressive loss of motor function. To determine if similar phenotypes are observed in *Drosophila*, we characterized mutations in *crowded-by-cid* (*cbc*), the *CLP1* ortholog, as well as in the fly ortholog of human *TSEN54*. Analyses of organismal viability, larval locomotion and brain size revealed that mutations in both *cbc* and *Tsen54* phenocopy those in mammals in several details. In addition to an overall reduction in brain lobe size, we also found increased cell death in mutant larval brains. Ubiquitous or tissue-specific knockdown of *cbc* in neurons and muscles reduced viability and locomotor function. These findings indicate that we can successfully model PCH in a genetically-tractable invertebrate.

## Abstract (precis)

**Summary:** Defects in the polynucleotide kinase, CLP1, and the tRNA splicing endonuclease (TSEN) complex display overlapping phenotypes in both vertebrates and invertebrates.

## INTRODUCTION

Transfer (t)RNAs play a crucial role in the heavily regulated process of protein expression. As highly structured adaptors, tRNAs translate nucleic acid messages into polypeptide outputs. In many organisms, including metazoans, most tRNA genes are intronless; their pre-tRNAs need only to be transcribed, end-processed, and modified before they can participate in protein translation (Phizicky and Hopper, 2010). However, a subset of tRNA genes contains introns (Chan and Lowe, 2009; 2016). These intervening sequences are generally small and interrupt the anticodon loop of the tRNA (Schmidt and Matera, 2020; Yoshihisa, 2014). Because tRNA structure is crucial for proper function, intron removal is a critically important step in tRNA biogenesis.

Metazoan tRNA intron removal is carried out in two enzymatic steps. First, a heterotetrameric enzyme complex, called TSEN, recognizes the pre-tRNA transcript (Paushkin et al., 2004). This complex cleaves the pre-tRNA in two places: at the 5′ exon-intron boundary, and at the intron-3′ exon boundary (Trotta et al., 1997). These cleavage events produce 2′,3′-cyclic phosphates on the 3′ ends of the 5′ exon and intron, and 5′-OH on the 5′ ends of the intron and 3′ exon (Abelson et al., 1998). Once the intron has been cut out of the pre-tRNA, a ligase enzyme, called RtcB, joins the two exon halves together using the cyclic phosphate on the 5′ exon as the junction phosphate to generate a mature tRNA (Popow et al., 2011). Strikingly, RtcB can only use the 5′-OH on the 3′ exon as a substrate for ligation; it cannot use a 5′-phosphate (Popow et al., 2011). In human and other animal cells, RtcB functions in a complex with other factors including archease and DDX1 (Popow et al., 2014); archease co-purifies with RtcB and cooperates with DDX1 to stimulate RtcB activity in biochemical assays (Popow et al., 2014). For many years, the fate of the excised metazoan tRNA intron was unknown. However, recent work from our lab has shown that, in animal cells, tRNA intron ends are ligated together to yield a unique subspecies of circular RNA, called a tRNA intronic circular RNA, or tricRNA (Lu et al., 2015; Schmidt and Matera, 2020; Schmidt et al., 2019). Earlier reports had shown that circularized tRNA introns (tricRNAs) from several archaeal species are generated in a similar manner (Salgia et al., 2003). Because this ligation event also utilizes the 2′,3′-cyclic phosphate as the junction phosphate, the overall process is termed the ‘direct ligation’ tRNA splicing pathway (Schmidt et al., 2019).

In contrast to direct ligation, plants and fungi are thought to utilize the ‘healing and sealing’ ligation pathway (Yoshihisa, 2014). Pre-tRNA cleavage is carried out by orthologs of the TSEN complex and the same non-canonical RNA ends are generated. However, the method of ligation is quite distinct. A multifunctional enzyme called Rlg1/Trl1 executes three activities: the 5′-OH of both the intron and 3′ exon is phosphorylated via its kinase domain; the 2′,3′-cyclic phosphate of the 5′ exon is opened through cyclic phosphodiesterase activity; and the exon halves are then joined by the ligase domain. The extra phosphate at the junction of the newly ligated tRNA is removed by a 2′-phosphotransferase enzyme called Tpt1 (Lopes et al., 2015). In contrast to the pathway employed by archaea and metazoa, the tRNA intron, now containing a 5′-phosphate, becomes a substrate for degradation by the exonuclease Xrn1 (Wu and Hopper, 2014).

Although there has been an extensive body of work on tRNA splicing in yeast and human cell culture models, less is known about the function of metazoan tRNA splicing enzymes *in vivo*. Among the many human neurological diseases, there is a class of disorders called Pontocerebellar Hypoplasia (PCH). Interestingly, several subytpes of PCH are associated with mutations in genes that encode the TSEN complex (Battini et al., 2014; Breuss et al., 2016; Budde et al., 2008; Cassandrini et al., 2010; Namavar et al., 2011a; 2011b; Valayannopoulos et al., 2012). Although the precise disease mechanisms are not well understood, each PCH subtype exhibits structural abnormalities in the brain, including microcephaly. These growth and tissue maintenance defects lead to developmental delays, mobility issues, and intellectual disabilities. Consistent with these reports in humans, RNAi-mediated knockdown of *Tsen54* in zebrafish embryos was shown to cause structural defects as well as cell death within the brain (Kasher et al., 2011). Further experimentation showed that genetic knockout of *Tsen54* was lethal in these animals (Kasher et al., 2011).

Recently, a new subtype of PCH was identified in several consanguineous families from eastern Turkey (Karaca et al., 2014; Schaffer et al., 2014; Wafik et al., 2018). PCH10 is associated with missense mutations in the human *CLP1* gene (Weitzer and Martinez, 2007). Originally identified as part of the mRNA 3′ end processing machinery (de Vries et al., 2000), the CLP1 polyribonucleotide 5′-hydroxyl kinase was subsequently found to copurify with the human TSEN complex, suggesting a role for this protein in pre-tRNA processing (Paushkin et al., 2004). Indeed, CLP1 can phosphorylate the 5′-ends of tRNA 3′ exons *in vitro* (Weitzer and Martinez, 2007). This finding was remarkable, considering that phosphorylation of the 5′-OH is known to inhibit RtcB-mediated ligation (Popow et al., 2011). We previously demonstrated that CLP1 is neither required for tRNA intron cleavage, nor for tRNA exon ligation (Hayne et al., 2020). Instead, CLP1 kinase activity serves as a negative regulator of tRNA processing, perturbing exon ligation as well as intron circularization (Hayne et al., 2020). Strikingly, all of the PCH10 cases reported to date document the same homozygous *CLP1* missense mutation: a G>A transition resulting in substitution of a histidine for an arginine residue (R140H) (Karaca et al., 2014; Schaffer et al., 2014; Wafik et al., 2018). This mutation has been shown to reduce the RNA kinase activity of CLP1 as well as its ability to associate with the TSEN complex (Karaca et al., 2014; Schaffer et al., 2014). Importantly, a Clp1 kinase-dead mouse model displayed similar neurodegenerative phenotypes to those of human PCH10 patients (Hanada et al., 2013).

Although tRNA disease-related phenotypes have been characterized in several vertebrate models, much less is known about tRNA processing in these systems, particularly the *in vivo* fate and status of tRNA introns. Due to the small size of vertebrate tRNA introns, most of which are less than 25 nt long (Chan and Lowe, 2016), they are difficult to detect. Given that human tRNA introns are very likely circularized (Schmidt et al., 2016), they would therefore evade typical sRNA sequencing approaches that depend on adaptor ligation prior to reverse transcription. These features make it difficult to study certain aspects of tRNA processing defects in vertebrate systems *in vivo*.

In contrast, the introns of several invertebrate tRNA genes are comparatively large (Lu et al., 2015). For example, one *Drosophila* tRNA gene (*CR31905*) contains a 113nt intron, and its resulting tricRNA can be easily detected throughout development by northern blotting and RNA sequencing (Lu et al., 2015). In addition, its topology (i.e. linear versus circular) can be verified by molecular biological assays (Lu et al., 2015). We also previously characterized tRNA processing factors in *Drosophila* cells, including Tsen54 (Schmidt et al., 2019) and crowded-by-cid (cbc), the fly ortholog of CLP1 (Hayne et al., 2020). Furthermore, there are sophisticated genetic tools available in flies that allow manipulation of gene expression in a very fine tissue-specific or developmental time-specific manner. In this study, we establish a *Drosophila melanogaster* model of PCH by characterizing available *Tsen54* and *cbc* mutants. We show that these animals exhibit viability, locomotor, and brain size phenotypes similar to those observed in PCH patients and vertebrate models. We also use RNA interference (RNAi) to determine if there is a tissue-specific requirement for cbc expression. In summary, this work establishes a new animal model of PCH wherein one can more readily examine tRNA splicing outputs.

## RESULTS

**Fig. 1.**
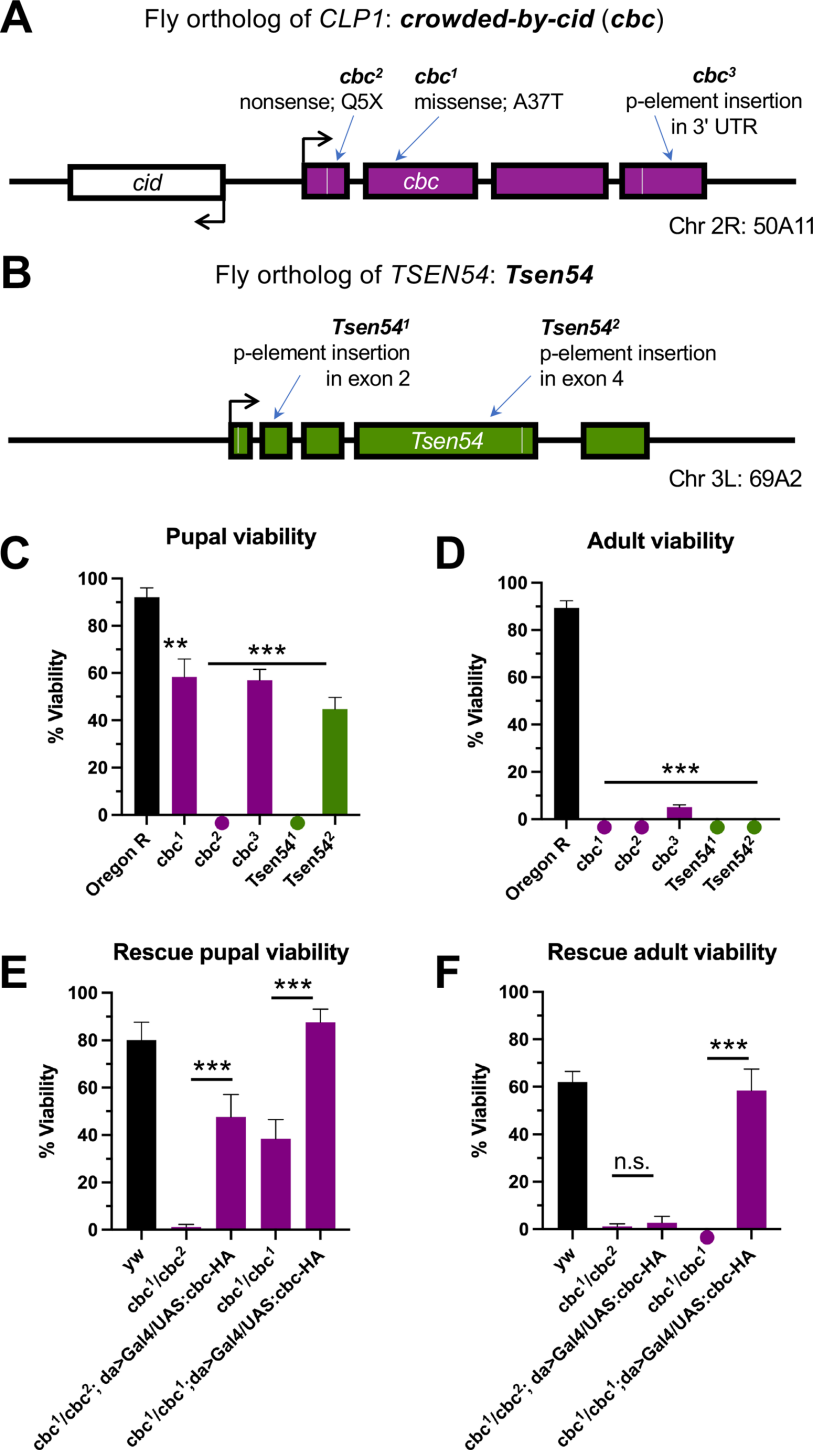
**Mutations in *Drosophila* tRNA processing factors cause strong viability defects.** (A,B) Summary of mutant alleles for *cbc* (A) and *Tsen54* (B). (C) Pupal and (D) adult average viability of homozygous mutants (*cbc* in purple, *Tsen54* in green) compared to control (Oregon R, in black). For each genotype, *n*=150 larvae were sorted into three vials (biological replicates) of 50 animals each, for three independent experiments. (E,F) Plot of the average pupal (E) and adult (F) viability of rescue crosses, as compared to those of the control (*yw*, in black). For each genotype, *n*=250 larvae were sorted into five vials (biological replicates) of 50 animals each, for five independent experiments. Note that, for certain mutants, a circle is placed below the *x*-axis to indicate a zero value rather than the absence of data. Error bars are SEM. **P<0.01; ***P<0.001, two-tailed Student's *t*-test. n.s., not significant.

Mutant lines used in this study were obtained from the Bloomington *Drosophila* Stock Center (see Table 1). As shown in Fig. 1A, the *cbc*^1^ and *cbc*^2^ alleles are EMS-induced mutations generated by the Kaufman lab that had been deposited at Bloomington, but never characterized (Larkin et al., 2021). The *cbc*^3^ allele is a p-element insertion in the 3′-UTR of *cbc*. We also obtained two p-element insertion alleles for *Tsen54*, called *Tsen54*^1^ and *Tsen54*^2^, corresponding to insertions in the second and fourth exons, respectively (Fig. 1B). Using these stocks, we set out to establish a tRNA splicing-based model of PCH in *Drosophila*.

**Table 1.**
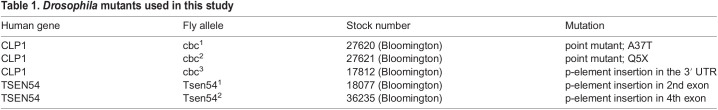
*Drosophila* mutants used in this study

### tRNA processing factor mutants display severe viability defects

We first examined viability in homozygous tRNA processing factor mutants using a standard assay. As shown in Fig. 1C, we identified significant defects in each of the mutants tested. Notably, the *cbc*^2^ allele was quite severe; these animals all die as embryos. Similarly, the *Tsen54*^1^ homozygotes die as third instar larvae. The remaining mutants (*cbc*^1^, *cbc*^3^, and *Tsen54*^2^) display pupation frequencies of ∼50% (Fig. 1C), and only the *cbc*^3^ homozygotes eclose as adults (albeit at a very low frequency, ∼5%; Fig. 1D).

We next tested whether the viability defects observed in the *cbc* mutants could be rescued by expression of wild-type *cbc*. Using a *daughterless*-Gal4 (da>Gal4) driver, we ubiquitously expressed a full-length, HA-tagged UAS:cbc transgene (Bischof et al., 2012) in the background of both *cbc*^1^*/cbc*^1^ homozygotes and *cbc*^1^*/cbc*^2^ trans-heterozygotes. Expression of wild-type cbc-HA protein significantly rescued pupal viability in both the homozygous and trans-heterozygous mutant backgrounds (Fig. 1E), and adult viability in the *cbc*^1^*/cbc*^1^ homozygotes (Fig. 1F). We note that the larvae expressing transgenic cbc-HA displayed prominent melanotic masses and that the adults that managed to eclose from these crosses all died within a few days. The melanotic masses are an innate immune response that is most likely caused by ectopic mis-expression of *cbc* in the wrong tissue and/or developmental stage. However, these data clearly demonstrate that the severe larval and pupal viability defects associated with the *cbc*^1^ (strong hypomorph) and *cbc*^2^ (presumptive null) alleles can be rescued by transgenic expression of cbc-HA.

This mutational analysis in flies is consistent with human PCH patient data, where one study reported the median age of death to be 50 months (Namavar et al., 2011a). Additionally, the observed viability defects are recapitulated in other animal models of PCH. For example, zebrafish bearing a homozygous nonsense mutation in *Tsen54* did not survive beyond 10 days post-fertilization (Kasher et al., 2011). Furthermore, *Clp1*-null mice die before embryonic day 6.5, a very early time point in mouse development (Hanada et al., 2013). We conclude that homozygous mutations in tRNA processing factors cause strong viability defects in *Drosophila*.

**Fig. 2.**
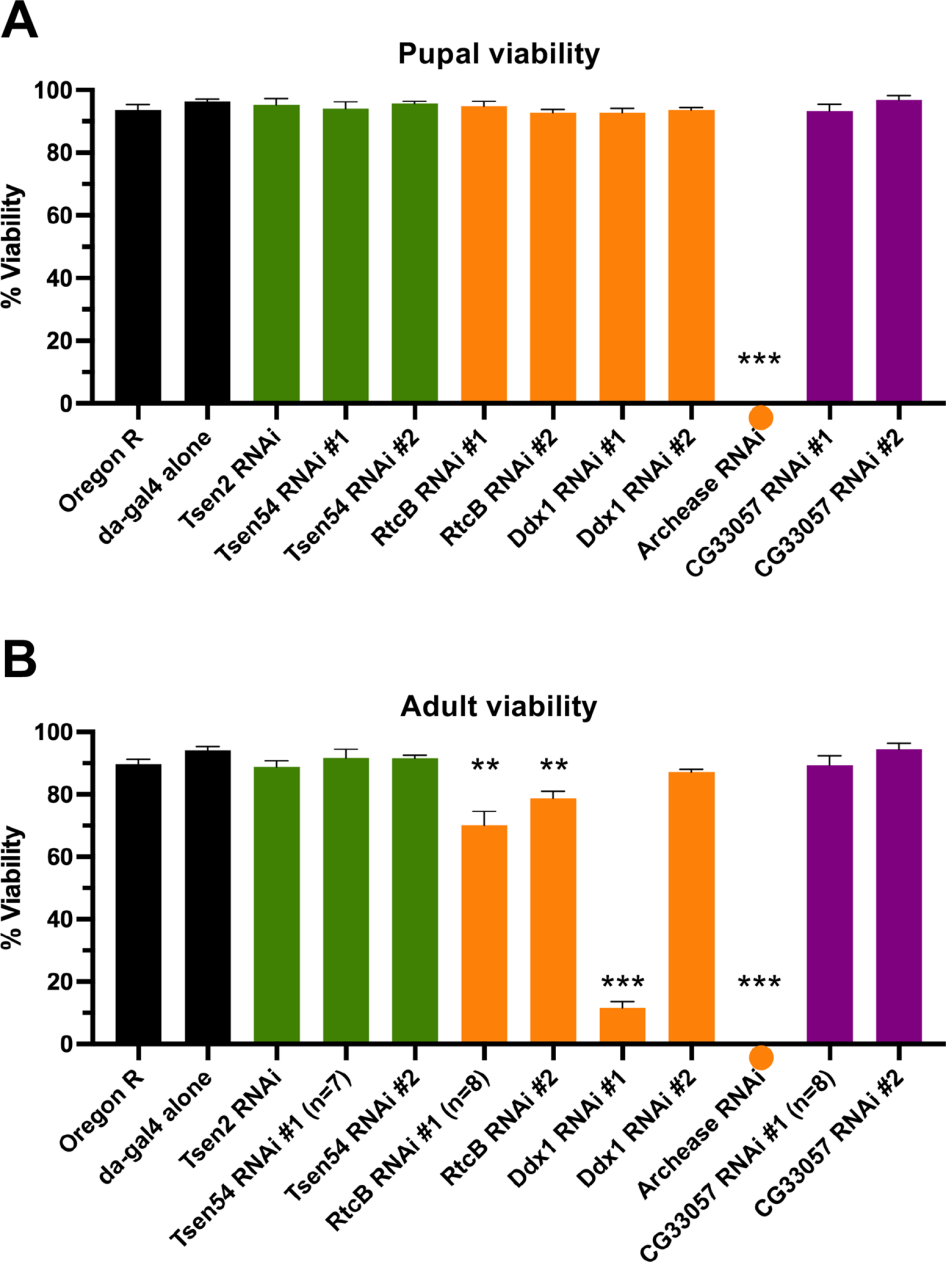
**Ubiquitous depletion of *Drosophila* tRNA ligation factors, but not cleavage factors, affects viability.** (A,B) Average pupation (A) and eclosion (B) frequencies for control and RNAi crosses. Oregon R and da-Gal4 alone (black) are controls. For each factor, only the da-Gal4×UAS:RNAi cross is shown (see Fig. S1 for UAS-RNAi alone pupal and adult viability). The green bars correspond to knockdown of pre-tRNA cleavage factors; the orange bars are for knockdown of tRNA ligation factors; and the purple bars represent the healing/sealing pathway factors. For each genotype, *n*=250 larvae were sorted into five vials (biological replicates) of 50 animals each, for five independent experiments [except where noted below graph; *n*=7 or eight biological replicates (350 or 400 larvae) for certain genotypes]. Error bars are SEM. **P<0.01, ***P<0.001, two-tailed Student's *t*-test. Note that a circle placed below the *x*-axis indicates a zero value rather than the absence of data.

We also assessed viability in animals depleted of known *Drosophila* tRNA processing factors (Schmidt et al., 2019) using RNAi. These transgenic constructs also utilize the Gal4-UAS system (Brand and Perrimon, 1993). We obtained UAS-RNAi lines for *Drosophila* tRNA processing factors (Table 2) and crossed these animals to either Oregon R (to control for the UAS-RNAi transgene) or to daughterless-Gal4 (to ubiquitously express shRNAs against the targeted tRNA processing factor). Additional controls for this experiment were Oregon R (wild type) and daughterless-Gal4 alone. None of the control crosses displayed pupal or adult (Fig. 2 and Fig. S1, black bars) viability defects. Interestingly, ubiquitous depletion of Tsen2, Tsen54, RtcB, and Ddx1 had no effect on pupation efficiency; however, animals expressing shRNA against archease failed to pupate and died as larvae (Fig. 2, orange dot). RNAi knockdown of Tsen2 or Tsen54 did not greatly affect adult viability (Fig. 2, green bars), which was interesting considering that depletion of these proteins strongly reduces tRNA and tricRNA production in a cellular model (Schmidt et al., 2019). This observation could be due to a relative dearth of transgenic expression, as has been observed for other shRNA constructs (Dietzl et al., 2007). Strikingly, we only observed significant adult viability defects upon depletion of RtcB ligase or other members of the ligation complex (Fig. 2, orange bars). As an additional control, we depleted *CG33057*, the putative *Drosophila* ortholog of the yeast 2′-phosphotransferase enzyme, Tpt1. Previously, we found that depletion of *CG33057/Tpt1* in a cell culture model had no effect on tricRNA formation (Schmidt et al., 2019). Here, we found that depletion of Tpt1 *in vivo* had no significant effect on pupal or adult viability (Fig. 2, purple bars). Overall, we observed a range of viability phenotypes when knocking down known tRNA processing factors in *Drosophila*.

**Table 2.**
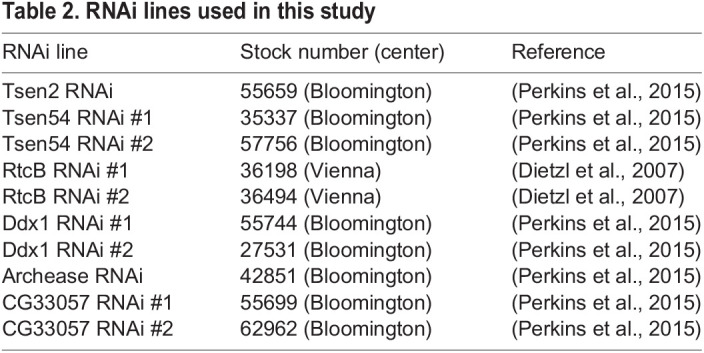
RNAi lines used in this study

### tRNA processing factor mutants exhibit larval locomotion defects

**Fig. 3.**
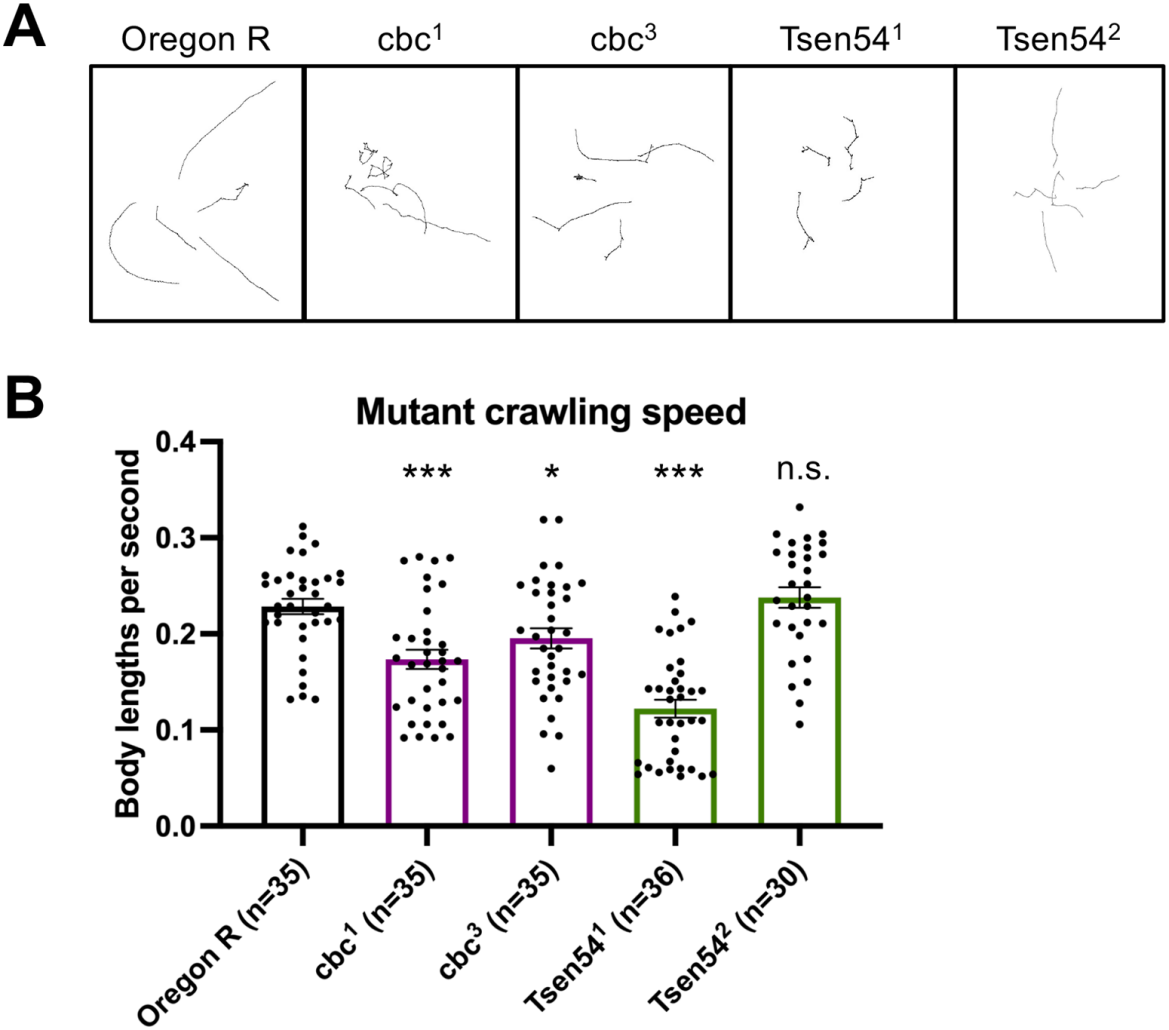
**tRNA processing factor mutants exhibit locomotion defects.** (A) For each genotype, five representative crawling paths are shown for third instar homozygous mutant or wild-type control larvae. (B) Bar plot of average crawling speed of third instar larvae, measured in body lengths per second. Each dot represents data from an individual larva. *n* values are listed for each genotype below the graph. Error bars are SEM. *P<0.05; ***P<0.001, two-tailed Student's *t*-test. n.s., not significant.

We next focused on mobility of the mutant animals. Previous reports from a Clp1-kinase dead knock-in mouse model found progressive locomotor defects in the homozygous mutants, including altered gait, reduced stride length, and impaired balance (Hanada et al., 2013). Furthermore, PCH patients are often reported to have a lack of motor development (Namavar et al., 2011b). To determine if the *Drosophila* mutants also displayed locomotor defects, we performed assays on wandering third instar larvae. All of the homozygous mutants except for *Tsen54*^2^ displayed a significant reduction in crawling speed, which is measured in body lengths per second to normalize for larval size (Fig. 3B). Interestingly, the locomotor defect was not quite as severe in the *cbc* mutants; these animals displayed crawling speeds that were more similar to wild type, although still significantly reduced. Representative crawling paths are shown in Fig. 3A. The wild-type animals crawled in relatively straight-line paths, whereas the motion paths of the mutants were typically shorter and had more turns. Overall, we observed locomotion defects in most of the mutants, consistent with both animal model and human patient data.

### tRNA processing factor mutants have reduced brain lobe size

**Fig. 4.**
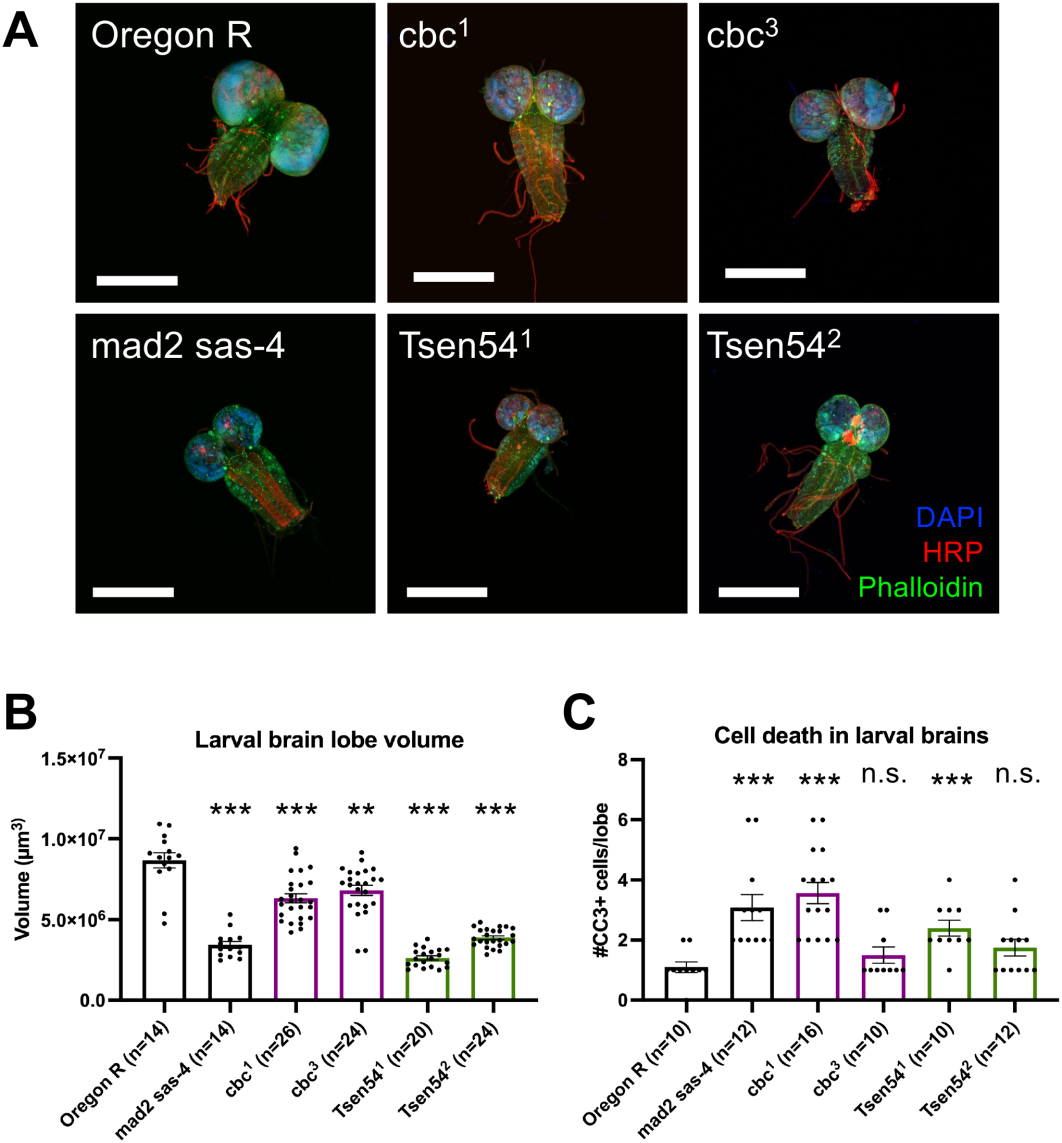
**tRNA processing factor mutants display significantly reduced brain lobe volume.** (A) Representative images of wandering third instar larval brains stained with anti-HRP, phalloidin-FITC, and DAPI. Scale bars: 300 µm. (B) Bar plot quantifying average larval brain lobe volume from animals analyzed in A. The controls are in black, *cbc* mutants are in purple, and *Tsen54* mutants are in green. Each dot represents data from an individual larval brain lobe. *n* values are listed below the graph for each genotype. Error bars are SEM. (C) Bar plot quantifying the average number of apoptotic cells per brain lobe. Genotypes and data analysis same as in panel B. **P<0.01, ***P<0.001, two-tailed Student's *t*-test. n.s., not significant.

The most prominent feature of PCH patients is microcephaly; the frequency of other phenotypes appears to be dependent on the specific disease subtype (Budde et al., 2008; Namavar et al., 2011b). Depletion of *Tsen54* in zebrafish embryos leads to brain hypoplasia as well as structural defects (Kasher et al., 2011). Clp1 kinase-dead mice exhibited both reduced brain weight and volume, as well as structural abnormalities (Hanada et al., 2013). Furthermore, zebrafish bearing a homozygous nonsense mutation in *Clp1* displayed gross head morphological defects and a massive increase in TUNEL-positive cells (Schaffer et al., 2014). To determine if the tRNA processing factor mutants recapitulate the brain phenotypes observed in mice, zebrafish, and human patients, we dissected third instar larval brains and performed immunofluorescence. Brains were stained with anti-HRP to detect neurons, phalloidin-FITC to detect actin, and DAPI to detect DNA. Imaging these stained brains revealed that all mutants displayed a reduction in brain lobe size (Fig. 4A, quantified in Fig. 4B), including a positive control known to exhibit microcephaly (Poulton et al., 2017). We quantified brain lobe volume as described by (Poulton et al., 2017) and found that the mutants and positive control showed a significant reduction in brain lobe volume.

We next tested whether the reduced volume was due to programmed cell death. Accordingly, we stained brains with an antibody against cleaved caspase 3 (CC3) to detect apoptotic cells (Fig. 4C). The positive control *mad2,sas-*4 double mutant displayed a significant increase in the number of CC3+ brain cells per lobe. Interestingly, the stronger of the two alleles for each processing factor (*cbc*^1^ and *Tsen54*^1^) both had a significant increase in apoptotic cells; however, the weaker of the two alleles (*cbc*^3^ and *Tsen54*^2^) did not show a significant difference in apoptotic cells, despite having significantly smaller brain lobes. Taken together, these data show that all tRNA processing factor mutants have significantly smaller brains than wild-type larvae, and this reduction in volume is due, at least in part, to apoptosis in the stronger mutants.

### Tissue-specific depletion of cbc causes viability and locomotion defects

**Fig. 5.**
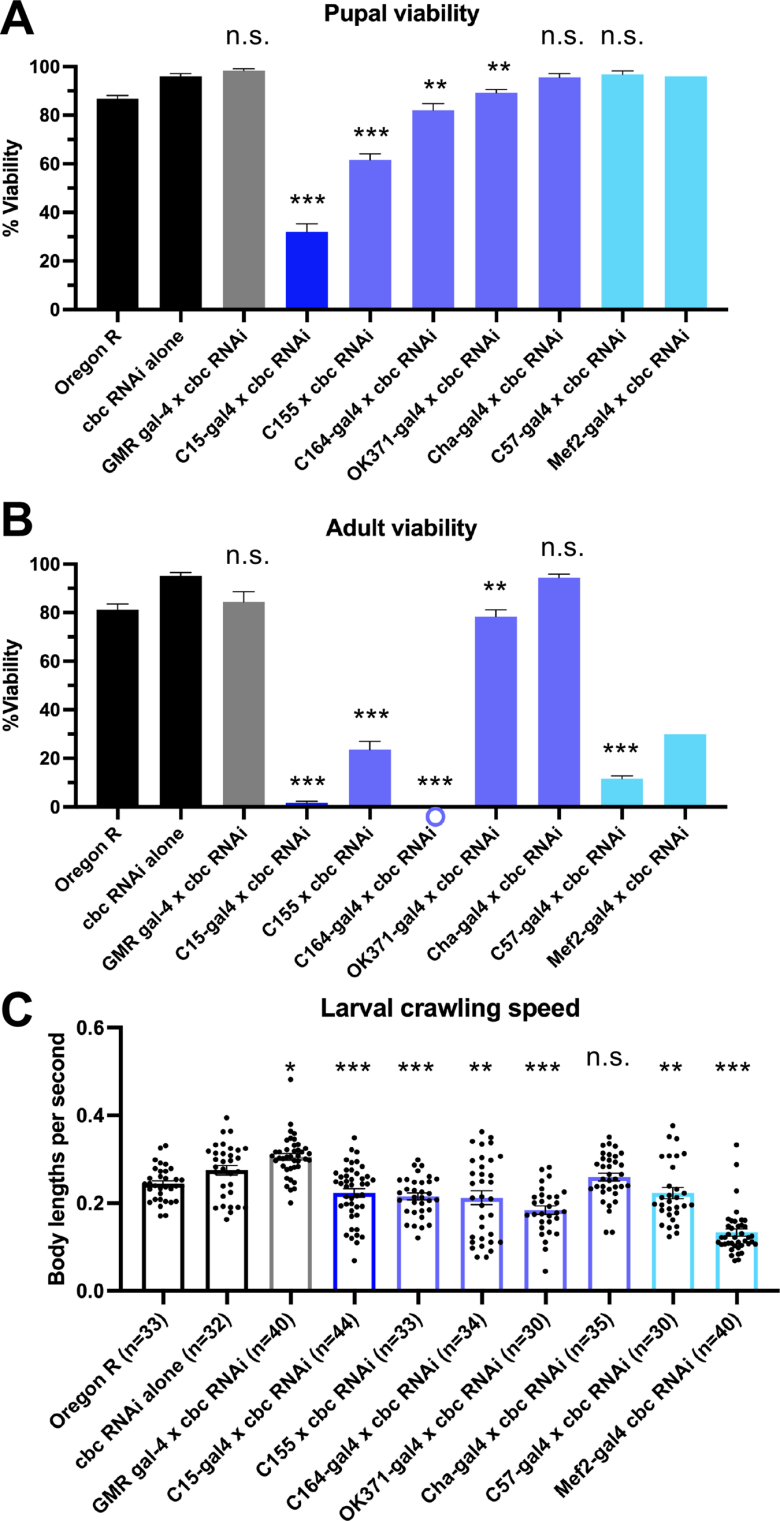
**Evidence for a tissue-specific requirement for cbc.** (A) Pupal and (B) adult average viability frequencies of control and RNAi crosses. Oregon R and cbc RNAi alone are negative controls. Each cross is listed across the *x*-axis (for data from Gal4 drivers alone, see Fig. S2). The gray bar is a control Gal4 driver, the dark blue bar is a combined neuron+muscle driver, purple bars are neuronal-only drivers, and light blue bars are muscle-specific drivers. For each genotype, *n*=250 larvae were sorted into five vials (biological replicates) of 50 animals each, for five independent experiments (except for Mef2, where *n*=50 larvae were sorted into one vial). Note that in some crosses a circle is placed below the *x*-axis to indicate a zero value rather than absence of data. Error bars are SEM. (C) Plot of average crawling speed for wandering third instar larvae in control and RNAi mutant crosses. Each dot represents data from an individual larva. Velocity is measured in body lengths per second. *n* values are listed for each genotype below the graph. Error bars are SEM. *P<0.05; **P<0.01; ***P<0.001, two-tailed Student's *t*-test. n.s., not significant.

Although tRNA splicing is a ubiquitous cellular process, certain tissues such as motor neurons seem to be more sensitive to mutations in tRNA processing enzymes. As evidence of a tissue-specific need for tRNA biogenesis, the neurodegenerative phenotypes observed in the Clp1 kinase-dead mice could be rescued by expression of wild-type Clp1 only in motor neurons via the Hb9 promoter (Hanada et al., 2013). To test if there is similar tissue specificity in flies, we knocked down *cbc* using various Gal4 drivers that target neurons and muscles (Table 3). As a control, we knocked down *cbc* in eye tissue (GMR-Gal4) and observed no strong defects in viability or locomotion (Fig. 5; see Fig. S2 for controls). We found the strongest developmental (Fig. 5A and B) and locomotor (Fig. 5C) defects using a combined neuron+muscle driver, C15-Gal4 (Spring et al., 2019). To further refine our analysis, we next used more specific neuron and muscle Gal4 drivers (Table 3). Depletion of *cbc* in all neurons (C155.L-Gal4) or motor neurons (C164-Gal4) had a strong effect on pupal and adult viability, as well as larval locomotion (Fig. 5). We observed weaker effects on viability and locomotion with glutamatergic (OK371-Gal4) or cholinergic (Cha-Gal4) neuron drivers (Fig. 5). Interestingly, although we did not observe any notable pupal viability defects using muscle-specific drivers (C57-Gal4 and Mef2-Gal4), depleting *cbc* from these tissues strongly reduced adult viability (Fig. 5A,B). Furthermore, *cbc* knockdown via Mef2-Gal4 severely affected larval crawling speed but C57-Gal4 did not (Fig. 5C). The expression patterns of the C57 and Mef2 drivers do not completely overlap in space and time (see Flybase for details). C57 is predominantly expressed in larval body wall muscles, whereas Mef2 is most prominent in myocytes but also drives expression in the fat body and certain neural tissues. Altogether, these results suggest that, consistent with findings in the mouse, cbc protein functions in motor neurons. However, we also show here that cbc is additionally important in muscles. Additional experiments are needed in order to fully elucidate the role(s) of cbc in these tissues.

**Table 3.**
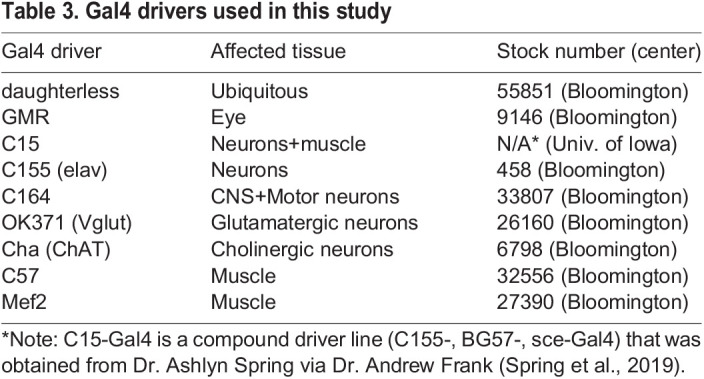
Gal4 drivers used in this study

## DISCUSSION

Genetic diseases that cause neuronal loss within the pons and cerebellum of the human brain typically do so early in development, and thus they were originally described as a form of underdevelopment, or hypoplasia (Namavar et al., 2011a). Subsequent studies revealed that pontocerebellar hypoplasias are more accurately described as a heterogeneous group of neurodegenerative disorders that typically present prenatally. Curiously, at least eleven different PCH subtypes have been described, and most of them are caused by mutations in genes that encode RNA processing factors, or regulators thereof (Van Dijk et al., 2018). The sporadic occurrence and heterogeneous presentation of rare genetic diseases like PCH make them quite difficult to study. In such cases, researchers turn to model systems to better understand these disorders.

Mutations in components of the *Drosophila* RNA exosome (Morton et al., 2020) have been shown to cause PCH-like phenotypes. This work represents the first report of tRNA splicing-based models of PCH in the fruitfly. Using available *cbc* and *Tsen54* mutants, we observed disease-related phenotypes in a genetically-tractable model organism. We also utilized ubiquitous and tissue-specific RNAi approaches to ascertain the *in vivo* function of tRNA splicing factors. Future refinements to these models should elucidate the molecular mechanisms by which the tRNA splicing machinery interfaces with metazoan neurological pathways and machineries.

It is important to note that our experiments were performed on available mutant lines from stock centers, rather than with specific patient-derived mutations. However, despite the lack of such specific mutations in our models, we observed PCH-related phenotypes in nearly all of the mutants tested. Strikingly, these animals all exhibited microcephaly (Fig. 4), similar to established PCH phenotypes (Battini et al., 2014; Breuss et al., 2016; Budde et al., 2008; Namavar et al., 2011a). Although tRNA splicing is implicated in PCH (Breuss et al., 2016; Karaca et al., 2014; Schaffer et al., 2014), it is possible that these diseases are caused by a non-tRNA splicing function of the TSEN complex or its presumptive regulatory factor, CLP1. In this case, one would need to generate patient-derived mutations in TSEN complex members or cbc. Due to the ease of genetic engineering in *Drosophila*, such disease-specific mutations, such as the R140H mutation in *CLP1*, would in theory be straightforward to make. The assays performed above could be repeated in these mutant animals to generate a more relevant disease model.

One benefit of using *Drosophila* as a model system is that we can directly examine tricRNAs in mutant and RNAi animals. Because tRNA and tricRNA biogenesis are affected in the same way by processing factor mutations (Schmidt et al., 2019), tricRNA levels can be used as a proxy for mature tRNAs, which can sometimes be difficult to detect due to their abundance, stability, and modifications. Based on previous work (Hayne et al., 2020; Schmidt et al., 2019), we predict that tricRNA abundance will be much lower in the *Tsen54* mutants. On the other hand, we hypothesize that the *cbc* mutants will have higher levels of tricRNAs than wild type. If cbc indeed functions as a kinase on tRNA 3′ exons and introns *in vivo*, RtcB would be unable use these 5′-phosphorylated molecules as substrates for ligation. Thus, mutation or depletion of cbc could remove this negative regulation of tRNA and tricRNA biogenesis. Northern blots of endogenous tricRNAs will reveal if these hypotheses are true. Such experiments would fit into a larger body of work on the link between tRNA processing and neurological disease, and perhaps provide insight into the mechanism of disease onset and progression.

## MATERIALS AND METHODS

### Fly lines and husbandry

We obtained *Tsen54* and *cbc* mutant alleles from the Bloomington *Drosophila* stock center (Table 1). The balancers for the *cbc* and *Tsen54* mutant lines were changed as follows: for the *cbc* alleles, the balancer was switched with the CyO-Actin-GFP second chromosome balancer; for the *Tsen54* alleles, the balancer was switched with the TM6B-GFP third chromosome balancer. We obtained UAS-RNAi lines from either the Bloomington or Vienna stock centers (Table 2). Gal4 drivers (Table 3) were obtained from the Bloomington stock center, with the exception of C15-Gal4 line, which was obtained from Dr. Ashlyn Spring, (see Spring et al., 2019 for details).

Stocks were maintained on standard molasses food. All fly stocks and crosses were performed at 25°, except for RNAi crosses which were performed at 29°. To generate homozygous mutant animals, males and females from a balanced stock were placed in a cage to mate, and females laid eggs on molasses plates with yeast paste. After allowing the plates to age for 1 day, GFP-negative larvae of mixed sex were sorted into vials for viability and locomotion assays (so as to reduce competition from heterozygous siblings). Rescue genotypes were generated in the same manner as mutant animals, except females laid eggs on grape juice plates rather than molasses plates.

### Viability assay

The viability assay was performed as described in Spring et al. (2019).

### Locomotor assay

The larval locomotion assay was performed as described in Spring et al. (2019) with the following modifications: videos were taken on an iPhone 8 and trimmed to exactly 45 s.

### Brain dissection and imaging

Wandering third instar larvae of mixed sex were gross dissected in PBS using sharpened forceps to expose the larval brain. The inverted carcasses were fixed in 4% formaldehyde, washed three times in PBT (0.1% Triton X-100 in PBS), blocked in PBT-G (1% normal goat serum+0.3% Triton X-100 in PBS) and incubated in primary antibody diluted in PBT-G overnight at 4°. The next day, the inverted carcasses were washed three times in PBT, incubated in secondary antibody+Phalloidin-FITC for one hour at 25°, and washed three additional times in PBS before fine dissecting the larval brains. The brains were mounted on a slide with mounting media containing DAPI. The slides were imaged using a Zeiss 710 confocal microscope, and the images were processed with ImageJ (https://imagej.nih.gov/ij/). The following antibodies were used in this study: rabbit anti-cleaved caspase 3 D175 at 1:100 (Cell Signaling Technology #9661); Alexa 647 goat anti-HRP at 1:500 (Jackson ImmunoResearch Laboratories Inc. 123-605-021); Alexa 488 goat anti-rabbit at 1:1000 (Life Technologies ref# A11008).

## Supplementary Material

Supplementary information
