# Supplementary material for: Mutations in Drosophila tRNA processing factors cause phenotypes similar to Pontocerebellar Hypoplasia
Source: Biol Open. 2022 Mar 18;11(3):bio058928. doi: 10.1242/bio.058928 (PMC8935212; doi:10.1242/bio.058928)
Supplement: Supplementary information [file biolopen-11-058928-s1.pdf]

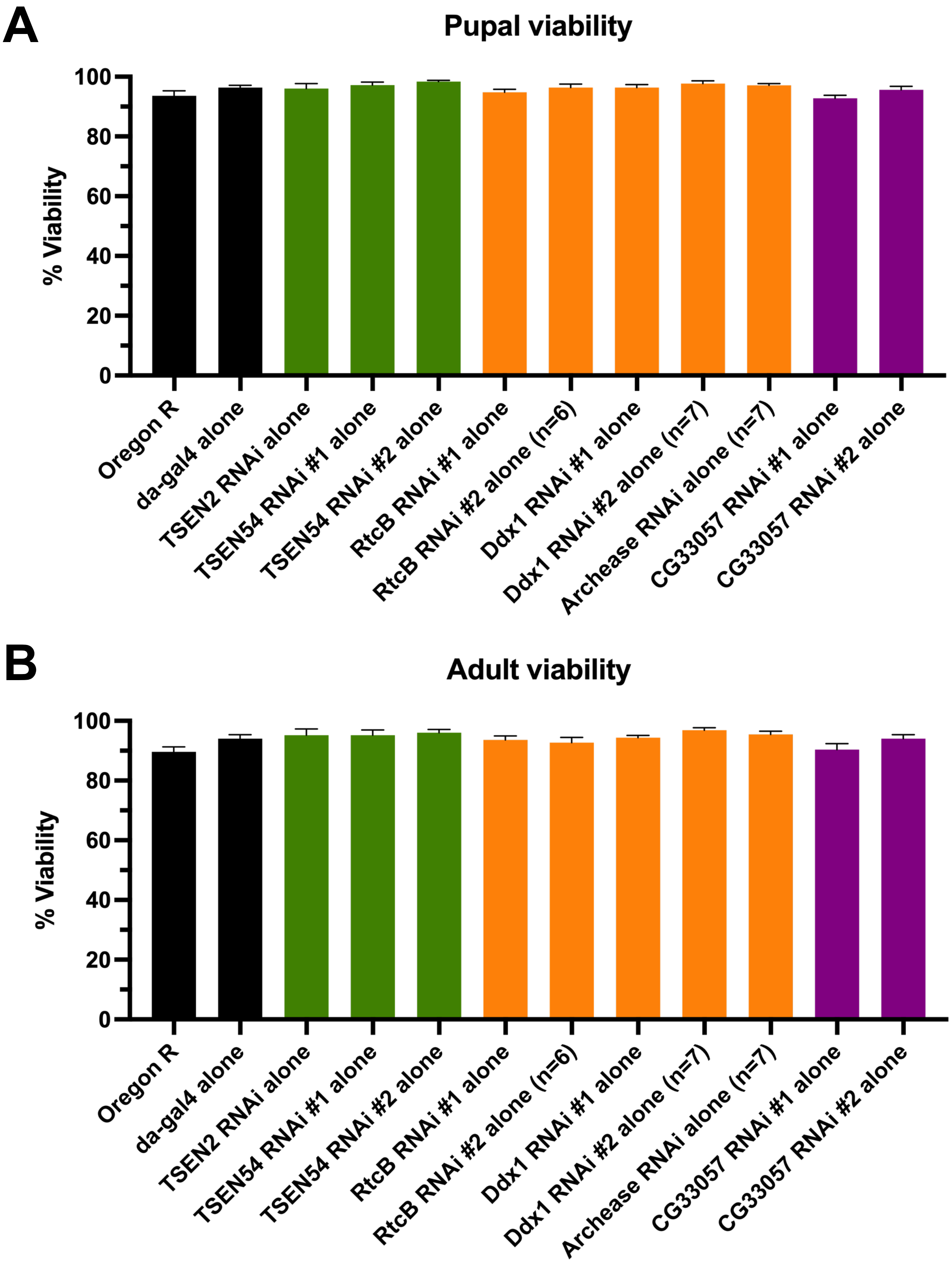
A
Pupal viability
% Viability
0
20
40
60
80
100
Oregon R
da-gal4 alone
TSEN2 RNAi alone
TSEN54 RNAi #1 alone
TSEN54 RNAi #2 alone
RtcB RNAi #1 alone
RtcB RNAi #2 alone (n=6)
Ddx1 RNAi #1 alone
Ddx1 RNAi #2 alone (n=7)
Archease RNAi alone (n=7)
CG33057 RNAi #1 alone
CG33057 RNAi #2 alone
B
Adult viability
% Viability
0
20
40
60
80
100
Oregon R
da-gal4 alone
TSEN2 RNAi alone
TSEN54 RNAi #1 alone
TSEN54 RNAi #2 alone
RtcB RNAi #1 alone
RtcB RNAi #2 alone (n=6)
Ddx1 RNAi #1 alone
Ddx1 RNAi #2 alone (n=7)
Archease RNAi alone (n=7)
CG33057 RNAi #1 alone
CG33057 RNAi #2 alone

Fig. S1. Expressing an RNAi element in the absence of gal4 does not affect animal viability.

(A) Pupal and (B) adult average viability of control RNAi crosses. Oregon R and da-Gal4 alone are in black. The green bars are pre-tRNA cleavage factors; the orange bars are tRNA ligation factors; and the purple bars are healing/sealing pathway factors. For each factor, only the UAS-RNAi element is expressed (and thus there should be no knockdown). For each genotype, n=250 larvae were sorted into 5 vials (biological replicates) of 50 animals each, for 5 independent experiments (except where noted; n=6 or 7 biological replicates (300 or 350 larvae) for certain genotypes). Error bars are SEM.

Biology Open • Supplementary information

A

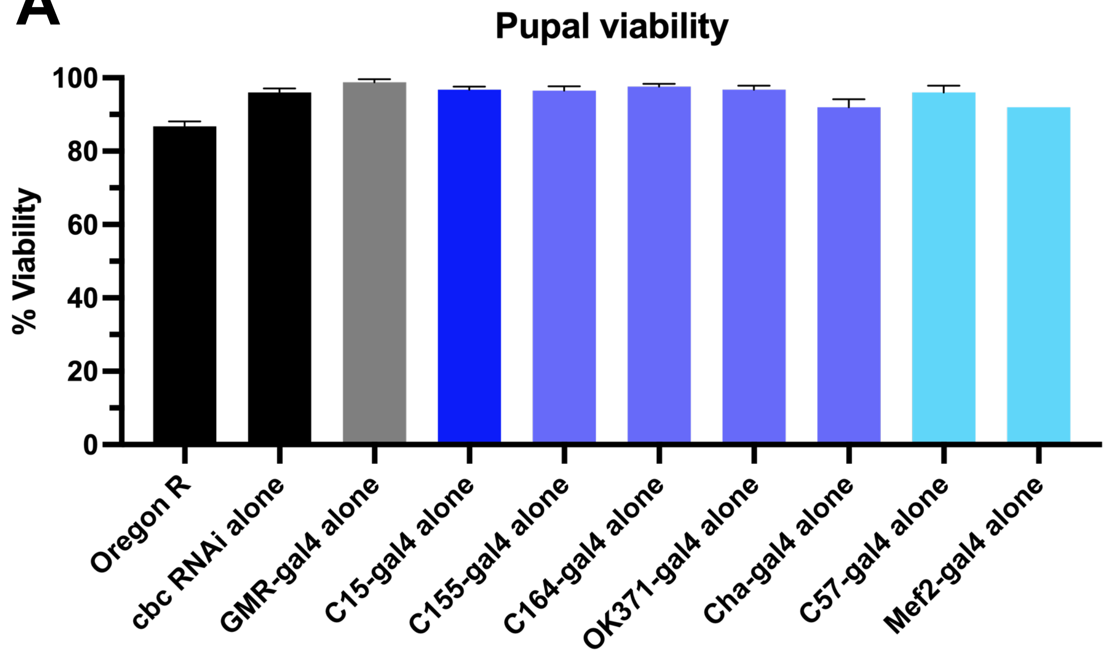

B

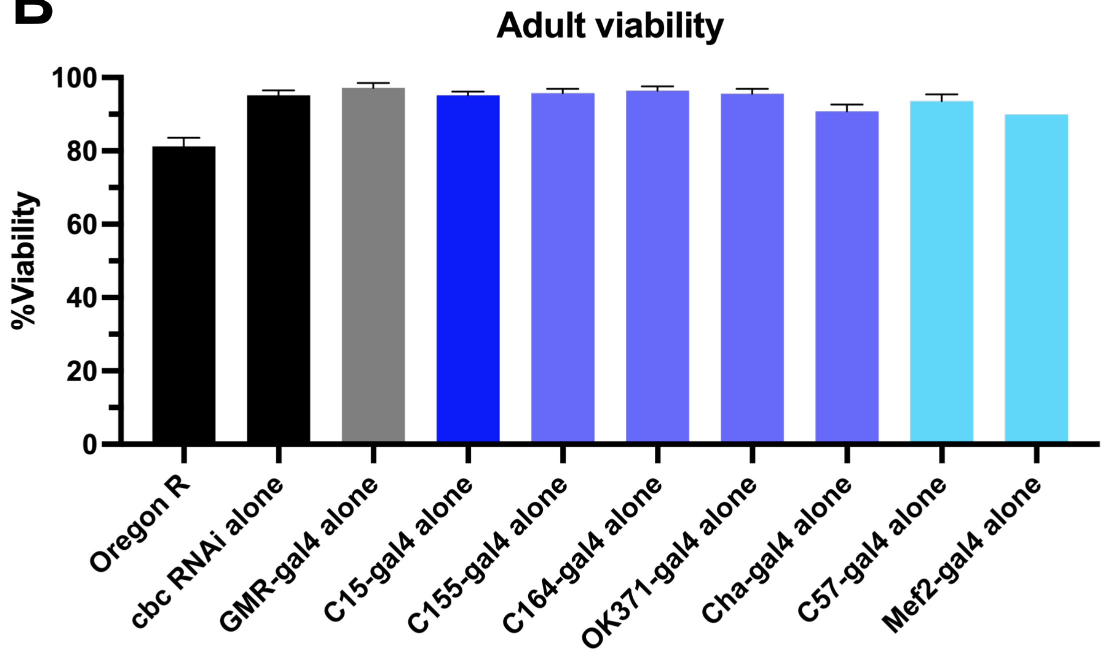

Biology Open • Supplementary information

Fig. S2. Tissue-specific gal4 drivers do not affect animal viability.

(A) Pupal and (B) adult average viability of various Gal4 drivers. Oregon R and cbc RNAi alone are in black. The gray bar is a control Gal4 driver, the dark blue bar is a combined neuron + muscle gal4 driver, purple bars are neuron gal4 drivers, and light blue bars are muscle gal4 drivers. For each genotype, n=250 larvae were sorted into 5 vials (biological replicates) of 50 animals each, for 5 independent experiments (except for Mef2, where n=50 larvae were sorted into 1 vial). Error bars are SEM.
